# Nutrition for Cancer Survivors

**DOI:** 10.3390/nu14194093

**Published:** 2022-10-01

**Authors:** Vassiliki Benetou

**Affiliations:** Department of Hygiene, Epidemiology and Medical Statistics, School of Medicine, National and Kapodistrian University of Athens, 11527 Athens, Greece; vbenetou@med.uoa.gr

Cancer is a leading cause of morbidity and mortality worldwide with tremendous burden for the individual and the society [1]. Fortunately, due to the earlier detection and better treatment for several cancer types, more people than ever before are surviving from cancer. In US, as of January 2022, it was estimated that 5.4% of the population were cancer survivors [2]. So, although the primary prevention of cancer is an absolute priority, it has become increasingly important to focus more on cancer survivors’ health and well-being.

Evidence has shown that nutrition plays an important role in primary prevention of cancer and currently evidence-based dietary recommendations exist, that if implemented, can substantially lower cancer risk [3]. Nevertheless, evidence on the role of diet after cancer diagnosis is limited. Cancer survivors are advised to follow, after the acute stage of their treatment, the dietary recommendations that apply for cancer prevention [3].

This Special Issue of Nutrients, “Nutrition for Cancer Survivors” aimed to elucidate further the association of nutrition and other nutrition-related factors among cancer survivors, with the goal to contribute in the development of evidence-based dietary recommendations and nutritional guidance tailored specifically for cancer survivors.

The Special Issue contains 11 original articles and two reviews on the topic.

The article by O’Callaghan and colleagues sets the scene for some very important questions [4]. Which are the dietary practices among cancer survivors? What are their food choices and quality of their diet? Are they satisfied with their food-related life? Which are the main diet-related concerns after surviving from cancer? An online survey, using validated dietary assessment tools, was implemented among cancer survivors in Ireland, during 2020, with the aim to explore these questions. The findings of the study emphasize, among others, the demand of cancer survivors to have individualized and specific nutritional advice and the need to integrate nutrition guidance into cancer care services.

This need was further addressed in two original articles by Keaver et al. [5,6]. The first study, a qualitative one, assessed the perceptions of oncology providers and cancer survivors in the US on the role of nutrition in cancer care, as well as their views on a new nutrition education program called “NutriCare” designed to be integrated into outpatient oncology care [5]. The second one presents a six-week pilot pretest-posttest intervention among breast cancer survivors implemented to test the feasibility and effectiveness of integrating the “NutriCare” program into practice [6]. The findings of both studies were encouraging since the majority of the participants found the program feasible, with a positive impact on their diet, whereas a significant improvement in their physical function was also noted after the intervention.

Pritlove et al. report the results of a pilot study of a nutritional intervention designed to address cancer-related fatigue (CRF), one of the most prevalent and distressing side effects experienced by cancer patients both during and after their treatment [7]. The Cooking for Vitality (C4V) is a culinary nutrition intervention program developed at the Princess Margaret Cancer Centre in Toronto, Canada. The goals of the program were to enhance participants’ understanding on how food choices can impact their energy intake levels, to establish basic food preparation and cooking skills, as well as culinary techniques that minimize the effort/energy required to prepare meals. The authors concluded that the C4V program was acceptable and helpful for cancer survivors and that the program may be effective in improving CRF levels, although a randomized controlled trial should confirm these results.

Cancer-related fatigue was also the outcome of interest in a phase II, nationwide, randomized controlled trial assessing the effects of fish vs. soybean oil supplementation on persistent CRF [8]. Kleckner et al. reported that the baseline nutritional status of cancer survivors, assessed with serum albumin, total lymphocytes, and blood cholesterol levels, plays an important role in CRF, since cancer survivors with good nutritional status had better uptake of fatty acid supplements and greater improvements in CRF in comparison to those with worse nutritional status.

The elucidation of the role of diet on cancer-related cognitive impairment was the goal of a cross-sectional feasibility study, implemented by Coro et al. with the use of online data from breast and colon cancer survivors in Australia [9]. Intake of fruits, vegetables, and long chain omega-3 polyunsaturated fatty acids (and its biomarker) was assessed, while self-reported and objective cognition status was measured by validated tools. Besides the investigation of an interesting research question, this study proposes a viable online/postal data collection method with high levels of participant engagement and satisfaction that may help other researchers as well.

The independent and joint associations of acid-producing diets and depression with physical health among 2944 breast cancer survivors, were investigated in the context of the Women’s Healthy Eating and Living (WHEL) study by Tessou et al. [10]. Increased dietary acid load and depression were each independently associated with reduced physical health, and jointly associated with worse physical health, assessed with the Rand Short Form-36 survey. Based on the results of this study, limiting the intake of acid-producing foods, such as processed meat, and not neglecting the importance of preserving cancer survivors’ mental health, seems crucial in the maintenance of their physical health.

Two of the included studies in this issue were implemented among breast cancer survivors undergoing therapy [11,12]. Mazzutti et al. focused-on women undergoing endocrine therapy with aromatase inhibitors and assessed, among others, their diet quality and intake, anthropometry, and physical activity levels as potential risk factors for future cardiovascular events [11]. The authors found a high percentage of diet inadequacy, both in quality and quantity, as well as high percentages of women being overweight, having abdominal obesity, and being physically inactive. The findings emphasize again the importance of integrating continuous nutrition and physical activity guidance into cancer survivors’ life. Santos et al. studied prospectively the nutritional status of women with breast cancer under chemotherapy in relation to inflammatory and antioxidant biomarkers [12]. The study highlights the importance of adopting healthier dietary practices, especially among overweight women, throughout their chemotherapy treatment.

The number of cancer survivors experiencing radiation-induced gastrointestinal symptoms has increased substantially during the last years in spite of the improved radiotherapy treatments. With the aim to reduce radiation-induced acute gastrointestinal symptoms, cancer patients are often advised to follow a low-fiber diet during radiotherapy. Animal studies can contribute to building evidence for possible causal associations. Thus, Patel et al. implemented a dietary intervention in male mice, both before and after their pelvic irradiation, evaluating the association of a high-oat bran diet vs. a no-fiber diet with pro-inflammatory cytokines [13]. The results indicate that fiber-rich oat-bran diet reduced the intensity of radiation-induced inflammation, both at the early and late stages. This finding may be considered in adjusting the current advice to consume a low-fiber diet during radiotherapy.

Elucidating the role of diet among cancer survivors from childhood cancer is also extremely important. Bérard et al. contributed to that end, by recording the dietary practices and exploring the associations of adherence to six a priori and a posteriori dietary patterns, as well as caloric intake from ultra-processed foods, with cardiometabolic indicators and inflammatory biomarkers, among survivors of acute lymphoblastic leukemia (ALL), participants in the PETALE study, implemented in Montreal, Canada [14].

Finally, two reviews are published in this Special Issue. Mann et al. reviewed possible mechanisms that diet could contribute to better prognosis among patients with colon, prostate, and breast cancer, patients with malignant gliomas, and patients under immunotherapy [15]. The review discusses findings mostly based on clinical studies and in vitro and in vivo animal models. Cava et al. conducted a narrative review among breast cancer survivors with a twofold aim: to summarize the evidence for the association of increased body weight with cancer recurrence and mortality, and to evaluate the effectiveness of interventions adhering to healthy dietary patterns to achieve normal body weight and reduce fat-related risks [16].

Overall, articles included in this Special Issue provide additional evidence on the role of diet and diet-related factors for cancer survivors, with the ultimate goal to contribute to the protection and promotion of health and well-being of this fasting growing population.

## References

[1] Sung H., Ferlay J., Siegel R.L., Laversanne M., Soerjomataram I., Jemal A., Bray F. (2021). Global Cancer Statistics 2020: GLOBOCAN Estimates of Incidence and Mortality Worldwide for 36 Cancers in 185 Countries. CA Cancer J. Clin..

[2] American Cancer Society (2022). Cancer Treatment & Survivorship Facts & Figures 2022–2024.

[3] World Cancer Research Fund/American Institute for Cancer Research Diet, Nutrition, Physical Activity and Cancer: A Global Perspective. Continuous Update Project Expert Report 2018. dietandcancerreport.org.

[4] O’Callaghan N., Douglas P., Keaver L. (2022). Nutrition Practices among Adult Cancer Survivors Living on the Island of Ireland: A Cross-Sectional Study. Nutrients.

[5] Keaver L., Yiannakou I., Folta S.C., Zhang F.F. (2020). Perceptions of Oncology Providers and Cancer Survivors on the Role of Nutrition in Cancer Care and Their Views on the “NutriCare” Program. Nutrients.

[6] Keaver L., Yiannakou I., Zhang F.F. (2020). Integrating Nutrition into Outpatient Oncology Care—A Pilot Trial of the NutriCare Program. Nutrients.

[7] Pritlove C., Capone G., Kita H., Gladman S., Maganti M., Jones J.M. (2020). Cooking for Vitality: Pilot Study of an Innovative Culinary Nutrition Intervention for Cancer-Related Fatigue in Cancer Survivors. Nutrients.

[8] Kleckner A.S., Culakova E., Kleckner I.R., Belcher E.K., Demark-Wahnefried W., Parker E.A., Padula G.D.A., Ontko M., Janelsins M.C., Mustian K.M. (2022). Nutritional Status Predicts Fatty Acid Uptake from Fish and Soybean Oil Supplements for Treatment of Cancer-Related Fatigue: Results from a Phase II Nationwide Study. Nutrients.

[9] Coro D.G., Hutchinson A.D., Dyer K.A., Banks S., Koczwara B., Corsini N., Vitry A., Coates A.M. (2022). ‘Food for Thought’—The Relationship between Diet and Cognition in Breast and Colorectal Cancer Survivors: A Feasibility Study. Nutrients.

[10] Tessou K.D., Lemus H., Hsu F.-C., Pierce J., Hong S., Brown L., Wu T. (2021). Independent and Joint Impacts of Acid-Producing Diets and Depression on Physical Health among Breast Cancer Survivors. Nutrients.

[11] Mazzutti F.S., Custódio I.D.D., Lima M.T.M., Carvalho K.P.d., Pereira T.S.S., Molina M.d.C.B., Canto P.P.L., Paiva C.E., Maia Y.C.d.P. (2021). Breast Cancer Survivors Undergoing Endocrine Therapy Have a Worrying Risk Factor Profile for Cardiovascular Diseases. Nutrients.

[12] Santos L.L.D., Custódio I.D.D., Silva A.T.F., Ferreira I.C.C., Marinho E.C., Caixeta D.C., Souza A.V., Teixeira R.R., Araújo T.G., Shivappa N. (2020). Overweight Women with Breast Cancer on Chemotherapy Have More Unfavorable Inflammatory and Oxidative Stress Profiles. Nutrients.

[13] Patel P., Malipatlolla D.K., Devarakonda S., Bull C., Rascón A., Nyman M., Stringer A., Tremaroli V., Steineck G., Sjöberg F. (2020). Dietary Oat Bran Reduces Systemic Inflammation in Mice Subjected to Pelvic Irradiation. Nutrients.

[14] Bérard S., Morel S., Teasdale E., Shivappa N., Hebert J.R., Laverdière C., Sinnett D., Levy E., Marcil V. (2020). Diet Quality Is Associated with Cardiometabolic Outcomes in Survivors of Childhood Leukemia. Nutrients.

[15] DO Mann S., DO Sidhu M., DO Gowin K. (2020). Understanding the Mechanisms of Diet and Outcomes in Colon, Prostate, and Breast Cancer; Malignant Gliomas; and Cancer Patients on Immunotherapy. Nutrients.

[16] Cava E., Marzullo P., Farinelli D., Gennari A., Saggia C., Riso S., Prodam F. (2022). Breast Cancer Diet “BCD”: A Review of Healthy Dietary Patterns to Prevent Breast Cancer Recurrence and Reduce Mortality. Nutrients.

